# Associations of total protein, albumin, and globulin with insulin resistance: an NHANES study

**DOI:** 10.3389/fendo.2024.1393137

**Published:** 2024-09-13

**Authors:** Rui-Xiang Zeng, Jun-Peng Xu, Yu-Zhuo Zhang, Jia-Wei Tan, Yong-Jie Kong, Min-Zhou Zhang, Li-Heng Guo

**Affiliations:** ^1^ The Second Clinical College of Guangzhou University of Chinese Medicine, Guangzhou, China; ^2^ Department of Critical Care Medicine, Guangdong Provincial Hospital of Chinese Medicine, Guangzhou, China

**Keywords:** total protein, albumin, globulin, insulin resistance, NHANES

## Abstract

**Objective:**

Insulin resistance (IR) is a well-established major risk factor for type 2 diabetes mellitus, nonalcoholic fatty liver disease, and atherosclerotic cardiovascular disease. Previous studies have shown an association between increased serum albumin (ALB) levels and the risk of IR. However, there is a lack of studies simultaneously evaluating the association of total protein (TP), ALB, and globulin (GLB) with IR.

**Methods:**

A total of 14,828 individuals (average age 49 ± 18 years) with complete data from the National Health and Nutrition Examination Survey (NHANES) were enrolled and divided into two groups (non-IR group, n = 8,653 and IR group, n = 6,175). Spearman’s correlation analysis, multivariable logistic regression models, restricted cubic spline curves, and subgroup analysis were performed to explore those associations.

**Results:**

After adjustment for potential confounders, multivariable logistic regression analysis revealed that scaled per 10g/L increment, the fully adjusted odds ratios (ORs) (95% confidence interval (CI)) for IR prevalence were 1.54 (95% CI 1.41-1.69, P < 0.0001), 1.09 (95% CI 0.95-1.25), P = 0.1995), and 1.62 (95% CI 1.47-1.79, P < 0.0001) for TP, ALB, and GLB respectively. Compared to those in the lowest quantiles, the prevalence of IR in subjects in the highest TP and GLB quantiles was 2.06 and 1.91 times, respectively. Furthermore, restrictive cubic curves confirmed that the relationship of TP, ALB, and GLB with IR prevalence was a linear relationship.

**Conclusions:**

The present cross-sectional study, for the first time, provided supportive evidence of positive associations of TP and GLB with IR, but not ALB, and demonstrated that TP and GLB might be useful markers for IR prevalence.

## Introduction

Insulin resistance (IR) is a medical condition that refers to a reduction in the responsiveness to insulin, promoting glucose uptake and utilization (1, 2). IR is a hallmark clinical feature of metabolic syndrome and a well-established major risk factor for type 2 diabetes mellitus (T2DM), nonalcoholic fatty liver disease, and atherosclerotic cardiovascular disease (ASCVD) (2–4). Furthermore, IR is a significant independent cardiovascular risk factor in non-diabetic subjects (5). Total protein (TP), comprising albumin (ALB) and globulin (GLB), is closely associated with various functions in the body, including not only malnutrition and immune function but also maintaining normal colloid osmotic pressure and pH balance, transporting various metabolites, and regulating the physiological effects of transported substances. The normal TP level typically ranges from 60 to 80 g/L, with ALB accounting for 35 to 50 g/L, while the remaining portion comprises GLB. Previous studies have demonstrated that increased serum ALB levels are related to several atherogenic risk factors including lipid profile, blood pressure, body mass index, and insulin resistance (6, 7). Furthermore, lower concentrations of serum ALB contribute to a higher risk of coronary heart disease (CHD), cardiovascular mortality, and carotid atherosclerosis (7–9). However, there is a paucity of studies simultaneously evaluating the relationship of TP, ALB, and GLB with IR. Therefore, the aim of the present study is to utilize data from large population-representative surveys to assess the concentrations of TP, ALB, and GLB associated with the prevalence of IR.

## Methods

### Study population

**Figure 1:**
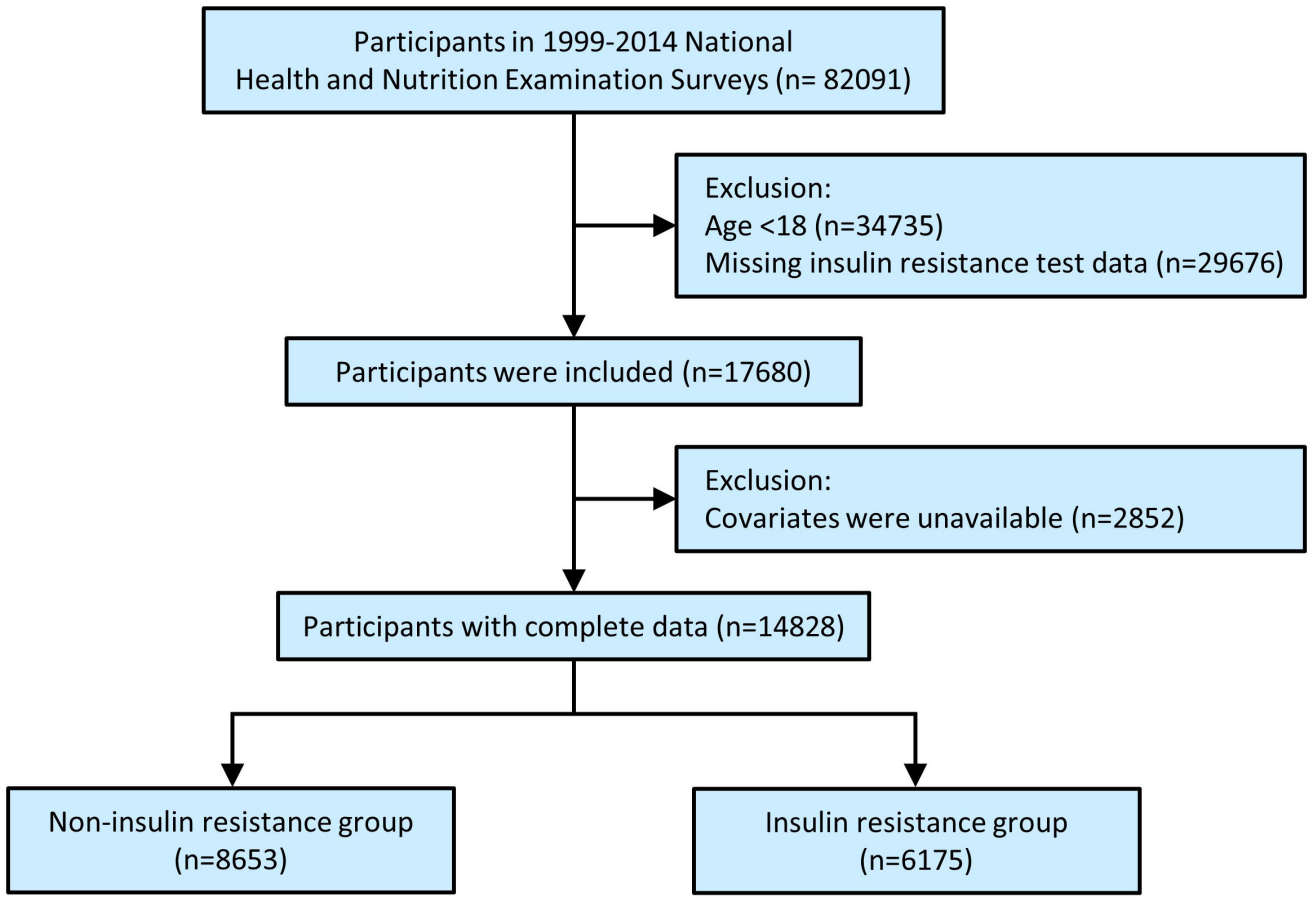
Study flowchart.

The National Health and Nutrition Examination Survey (NHANES) is a series of national surveys to evaluate the health status of the population with a complex stratified multistage probability sampling method. The United States National Center for Health Statistics (Centers for Disease Control and Prevention, Atlanta, GA, USA) ratified the study protocols, and all the participants provided written informed consent. Details about the NHANES have been published elsewhere (10, 11). The present population-based study enrolled participants with publicly available data from NHANES 1999 to 2014. As shown in **Figure 1**, the total number of participants in the primary survey was 82,091. After excluding participants who were aged < 18 years (n = 34,735), who were missing baseline IR test data (n = 29,676), and for which covariates were unavailable (n = 2,852), 14,828 individuals with complete data were enrolled in the final analysis and divided into two groups: the non-IR group (n = 8,653) and the IR group (n = 6,175).

### Covariate information

Fasting samples obtained from peripheral venous blood were stored at a maximum of −20°C and shipped weekly for laboratory analyses. Fasting-blood-glucose (FBG) was analyzed using a Hexokinase-mediated reaction (Roche/Hitachi Modular P Chemistry Analyzer). Fasting glucose-insulin (FIN) was measured by the Merocodia Insulin ELISA, which is a two-site enzyme immunoassay utilizing the direct sandwich technique. In the present study, IR was defined as homeostasis model assessment (HOMA)-IR ≥ 2.73 according to previous studies in American adults (12–14). HOMA-IR was calculated as the FIN (μU/mL) × FBG (mmol/L)/22.5 (15).

The concentration of TP was measured on the DxC800 Synchro (Beckman Coulter UniCel) by the timed rate biuret method, and the bichromatic digital endpoint method was used to measure the ALB concentration. GLB levels were calculated from TP minus ALB levels. Levels of serum creatinine (Scr), hemoglobin (Hb), glycated hemoglobin A1c (HbA1c), and lipid profiles were tested and recorded in authoritative laboratories using standard procedures.

Demographic variables were acquired according to the household interview, such as age, gender, race, and education. Information on smoking status and history of disease had been assessed at baseline by standard examinations and questionnaires by trained health technicians, interviewers, and physicians. The mean blood pressure was calculated as an average of three valid measurements. Detailed analysis methods can be accessed on the NHANES website (https://www.cdc.gov/nchs/nhanes/index.htm).

### Statistical analysis

Baseline characteristics of the included participants were divided by IR or not (non-IR group and IR group). The data were presented as mean values with standard deviation (SD), the median with interquartile ranges, or frequencies with percentages, as appropriate. Comparisons of the differences between groups were made with one-way ANOVA, chi-square tests, or Kruskal-Wallis H-tests by IR or not. The heatmap of the correlation between covariates used Spearman’s correlation analysis. In analyses examining associations with IR incidence, TP, ALB, and GLB were treated as continuous independent variables, scaled per 10g/L increment, or divided into six groups, using multivariable logistic regression models with different adjustments to calculate the odds ratios (ORs) and corresponding 95% confidence intervals (CIs). In model 1, there was no adjustment. In model 2, we adjusted for age, gender, and race. In model 3, we adjusted for age, gender, race, education, systolic blood pressure, diastolic blood pressure, body mass index, smoking, diabetes, hypertension, coronary heart disease, acute myocardial infarction, chronic heart failure, stroke, cancer, alanine aminotransferase (ALT), aspartate aminotransferase (AST), Scr, Hb, HbA1c, triglycerides (TG), total cholesterol (TC), and high-density lipoprotein cholesterol (HDL-C). Restricted cubic spline models were used for nonlinear relationships with knots at the 5th, 35th, 65th, and 95th percentiles of TP, ALB, or GLB. If the relationships were non-linear, the difference in relationships at the threshold was detected by two piecewise linear regression models. The point with the highest likelihood among all the possible values was chosen to define the threshold value. Furthermore, several subgroup analyses were performed, including age (<65 or ≥65 years), gender (man or woman), race (white or Black), BMI (<25 or ≥25 kg/m^2^), smoking (non-smoker or smoker), diabetes (yes or no), coronary heart disease (yes or no), acute myocardial infarction (yes or no), chronic heart failure (yes or no), stroke (yes or no), and cancer (yes or no). All statistical analyses were performed using R version 3.6.1 (R Foundation for Statistical Computing, Vienna, Austria), and P < 0.05 was considered statistically significant.

## Results

### Baseline characteristics

**Table 1** demonstrates the baseline characteristics of the 14,828 participants (average age 49 ± 18 years), including 8,653 without IR and 6,175 IR cases. Overall, there were significant differences in the baseline characteristics between the non-IR and IR groups. Compared to the non-IR group, participants with IR were older and had more comorbidities, such as diabetes, hypertension, coronary heart disease, acute myocardial infarction, chronic heart failure, stroke, and cancer, with higher levels of systolic/diastolic blood pressure, BMI, ALT, AST, Scr, Hb, HbA1c, TG, TC, and HDL-C.

**Table 1:** Baseline clinical and procedural characteristics.

Variable	Overall	Non-IR	IR	P-value
	(n = 14828)	(n = 8653)	(n = 6175)	
Age, years	49 ± 18	47 ± 18	51 ± 17	<0.001
Males	7639 (51.52%)	4619 (53.38%)	3020 (48.91%)	<0.001
Race				<0.001
White	7169 (48.35%)	4547 (52.55%)	2622 (42.46%)	
Black	2830 (19.09%)	1515 (17.51%)	1315 (21.30%)	
Other	4829 (32.57%)	2591 (29.94%)	2238 (36.24%)	
Education				<0.001
Lower than high school	4273 (28.82%)	2224 (25.70%)	2049 (33.18%)	
High school	3424 (23.09%)	1955 (22.59%)	1469 (23.79%)	
More than high school	7131 (48.09%)	4474 (51.70%)	2657 (43.03%)	
Smoking				<0.001
Never Smoker	7859 (53.00%)	4606 (53.23%)	3253 (52.68%)	
Current Smoker	3160 (21.31%)	1985 (22.94%)	1175 (19.03%)	
Ex-Smoker	3809 (25.69%)	2062 (23.83%)	1747 (28.29%)	
SBP, mmHg	124 ± 19	121 ± 20	126 ± 18	<0.001
DBP, mmHg	70 ± 12	69 ± 12	71 ± 13	<0.001
BMI, kg/m^2^	28.59 ± 6.34	26.05 ± 4.78	32.15 ± 6.53	<0.001
Diabetes	1571 (10.59%)	408 (4.72%)	1163 (18.83%)	<0.001
Hypertension	4913 (33.13%)	2170 (25.08%)	2743 (44.42%)	<0.001
Coronary heart disease	586 (3.95%)	258 (2.98%)	328 (5.31%)	<0.001
AMI	611 (4.12%)	274 (3.17%)	337 (5.46%)	<0.001
Chronic heart failure	422 (2.85%)	176 (2.03%)	246 (3.98%)	<0.001
Stroke	490 (3.30%)	233 (2.69%)	257 (4.16%)	<0.001
Cancer	1302 (8.78%)	726 (8.39%)	576 (9.33%)	0.047
Total protein, g/L	72.30 ± 5.04	71.99 ± 5.03	72.74 ± 5.03	<0.001
Albumin, g/L	42.21 ± 3.60	42.51 ± 3.63	41.77 ± 3.50	<0.001
Globulin, g/L	30.10 ± 4.58	29.48 ± 4.40	30.97 ± 4.68	<0.001
ALT, u/L	21 (16–28)	19 (15–25)	24 (18–33)	<0.001
AST, u/L	23 (19–27)	22 (19–26)	23 (20–28)	<0.001
Scr, mg/dl	72 (61–88)	71 (62–88)	73 (62–88)	<0.001
Hemoglobin, g/L	14.25 ± 1.56	14.19 ± 1.53	14.34 ± 1.59	<0.001
HbA1c, %	5.65 ± 1.04	5.39 ± 0.64	6.02 ± 1.34	<0.001
TG,mg/dl	107 (74–161)	91 (65–132)	137 (95–198)	<0.001
TC,mg/dl	199 ± 42	198± 42	200 ± 43	0.01
HDL-C,mg/dl	54 ± 16	58 ± 16	48 ± 13	<0.001
FBG, mmol/L	5.60 ± 1.89	5.04 ± 0.92	6.39 ± 2.52	<0.001
Fasting insulin, μU/mL	9.77 (6.32-15.91)	6.87 (4.92-8.95)	17.61 (13.83-24.38)	<0.001
HOMA-IR	2.30 (1.41-4.01)	1.54 (1.08-2.04)	4.52 (3.41-6.68)	<0.001

IR, insulin resistance; SBP, systolic blood pressure; DBP, diastolic blood pressure; BMI, body mass index; AMI, acute myocardial infarction; ALT, alanine aminotransferase; AST, aspartate aminotransferase; Scr, serum creatinine; HbA1c, glycated hemoglobin A1c; TG, triglycerides; TC, total cholesterol; HDL-C, high-density lipoprotein cholesterol; FBG, fasting blood-glucose; HOMA-IR, homeostasis model assessment of insulin resistance.

Values are expressed as the mean ± SD, the median with interquartile range or n (%).

### Relationship of TP, ALB, and GLB with incident IR

**Figure 2:**
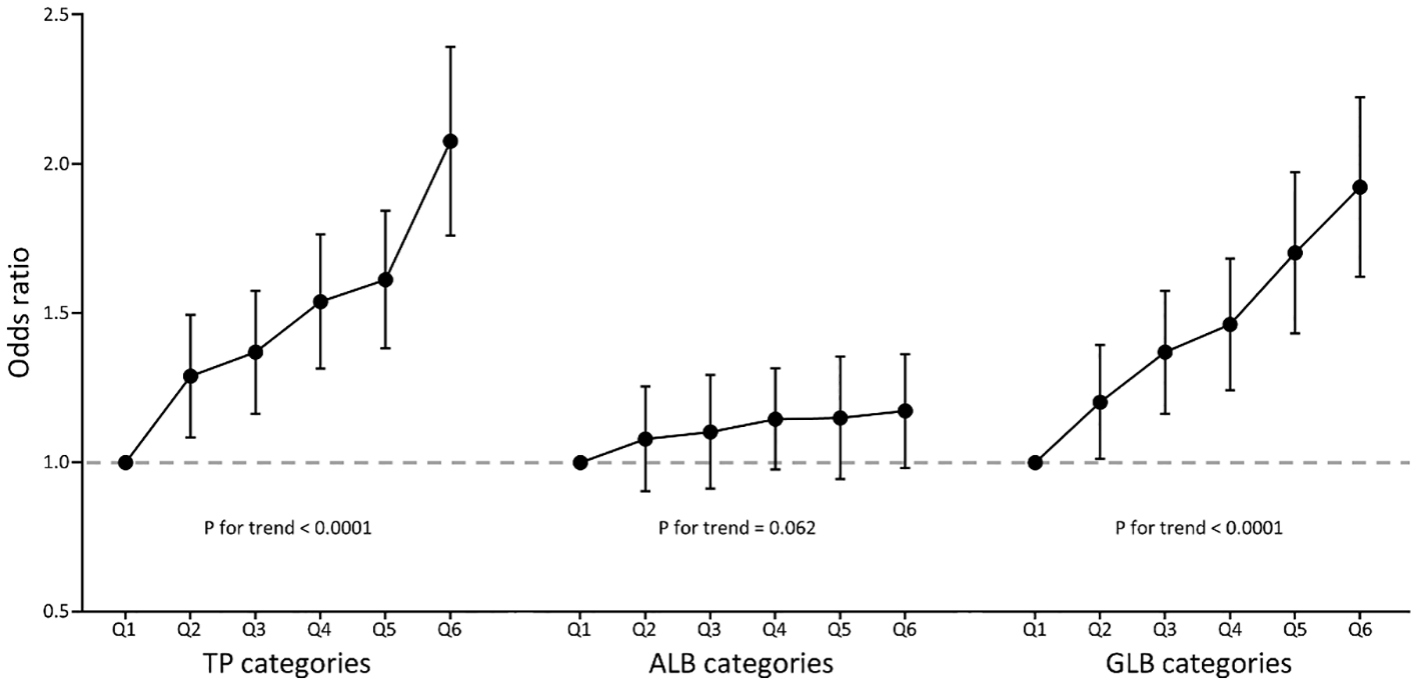
Multivariate-adjusted odds ratio (95% CI) of TP, ALB, and GLB with insulin resistance in categories analyses. TP, total protein; ALB, albumin; GLB, globulin. Multivariate model adjusted for age, gender, race, education, systolic blood pressure, diastolic blood pressure, body mass index, smoking, diabetes, hypertension, coronary heart disease, acute myocardial infarction, chronic heart failure, stroke, cancer, alanine aminotransferase, aspartate aminotransferase, serum creatinine, glycated hemoglobin A1c, hemoglobin, total cholesterol, high-density lipoprotein cholesterol, and triglycerides.

The multivariate logistic regression results are summarized in **Table 2**. In model 3, when TP, ALB, or GLB was treated as a continuous variable, and scaled per 10g/L increment, the fully adjusted ORs (95% CI) for Incident IR were 1.54 (95% CI 1.41-1.69, P < 0.0001), 1.09 (95% CI 0.95-1.25), P = 0.1995), and 1.62 (95% CI 1.47-1.79, P < 0.0001) for TP, ALB, and GLB respectively. When TP, ALB, or GLB were treated as a categorical variable, the effects of TP and GLB in increasing the risks of IR were positive (all P for trend < 0.0001), but the effect of ALB was not (P for trend = 0.062). Compared to those in the lowest quantiles, the prevalence of IR for subjects in the highest TP and GLB quantiles was 2.06 and 1.91 times, respectively. The ORs of TP, ALB, and GLB for incident IR are shown in **Figure 2**, indicating the linear relationships in TP and GLB.

**Table 2:** Relationships of TP, ALB, and GLB with insulin resistance.

	Odds ratio (95% CI), P-value		
	Model 1	Model 2	Model 3
**TP (per 10g/L increment)**	1.34 (1.26, 1.43) <0.0001	1.21 (1.13, 1.29) <0.0001	1.54 (1.41, 1.69) <0.0001
TP categories			
Q1	Reference	Reference	Reference
Q2	1.29 (1.13, 1.46) <0.0001	1.21 (1.07, 1.38) 0.0032	1.28 (1.09, 1.50) 0.0025
Q3	1.32 (1.17, 1.48) <0.0001	1.23 (1.09, 1.39) 0.0008	1.36 (1.17, 1.58) <0.0001
Q4	1.51 (1.34, 1.70) <0.0001	1.38 (1.22, 1.55) <0.0001	1.53 (1.32, 1.77) <0.0001
Q5	1.46 (1.31, 1.64) <0.0001	1.32 (1.17, 1.48) <0.0001	1.60 (1.39, 1.85) <0.0001
Q6	1.65 (1.47, 1.84) <0.0001	1.40 (1.25, 1.58) <0.0001	2.06 (1.77, 2.40) <0.0001
P for trend	<0.0001	<0.0001	<0.0001
**ALB (per 10g/L increment)**	0.56 (0.51, 0.62) <0.0001	0.52 (0.47, 0.57) <0.0001	1.09 (0.95, 1.25) 0.1995
ALB categories			
Q1	Reference	Reference	Reference
Q2	1.03 (0.91, 1.17) 0.6052	0.95 (0.84, 1.08) 0.4363	1.07 (0.91, 1.26) 0.3993
Q3	0.90 (0.79, 1.03) 0.1345	0.81 (0.70, 0.92) 0.0021	1.09 (0.92, 1.30) 0.3117
Q4	0.77 (0.69, 0.86) <0.0001	0.69 (0.62, 0.78) <0.0001	1.14 (0.98, 1.32) 0.0927
Q5	0.72 (0.63, 0.82) <0.0001	0.64 (0.56, 0.74) <0.0001	1.14 (0.95, 1.36) 0.1544
Q6	0.55 (0.49, 0.61) <0.0001	0.50 (0.44, 0.56) <0.0001	1.16 (0.99, 1.37) 0.0653
P for trend	<0.0001	<0.0001	0.062
**GLB (per 10g/L increment)**	2.08 (1.93, 2.24) <0.0001	1.93 (1.78, 2.10) <0.0001	1.62 (1.47, 1.79) <0.0001
GLB categories			
Q1	Reference	Reference	Reference
Q2	1.26 (1.11, 1.44) 0.0004	1.26 (1.10, 1.43) 0.0006	1.19 (1.02, 1.40) 0.0275
Q3	1.57 (1.39, 1.78) <0.0001	1.54 (1.36, 1.74) <0.0001	1.36 (1.17, 1.58) <0.0001
Q4	1.84 (1.63, 2.08) <0.0001	1.78 (1.57, 2.02) <0.0001	1.45 (1.25, 1.69) <0.0001
Q5	2.22 (1.95, 2.52) <0.0001	2.13 (1.86, 2.43) <0.0001	1.69 (1.44, 1.98) <0.0001
Q6	2.77 (2.45, 3.12) <0.0001	2.54 (2.23, 2.88) <0.0001	1.91 (1.63, 2.23) <0.0001
P for trend	<0.0001	<0.0001	<0.0001

TP, total protein; ALB, albumin; GLB, Globulin.

Model 1: no adjustment; Model 2: adjusted for age, gender, and race; Model 3: adjusted for age, gender, race, education, systolic blood pressure, diastolic blood pressure, body mass index, smoking, diabetes, hypertension, coronary heart disease, acute myocardial infarction, chronic heart failure, stroke, cancer, alanine aminotransferase, aspartate aminotransferase, serum creatinine, glycated hemoglobin A1c, hemoglobin, total cholesterol, high-density lipoprotein cholesterol, triglycerides.

**Figure 3:**
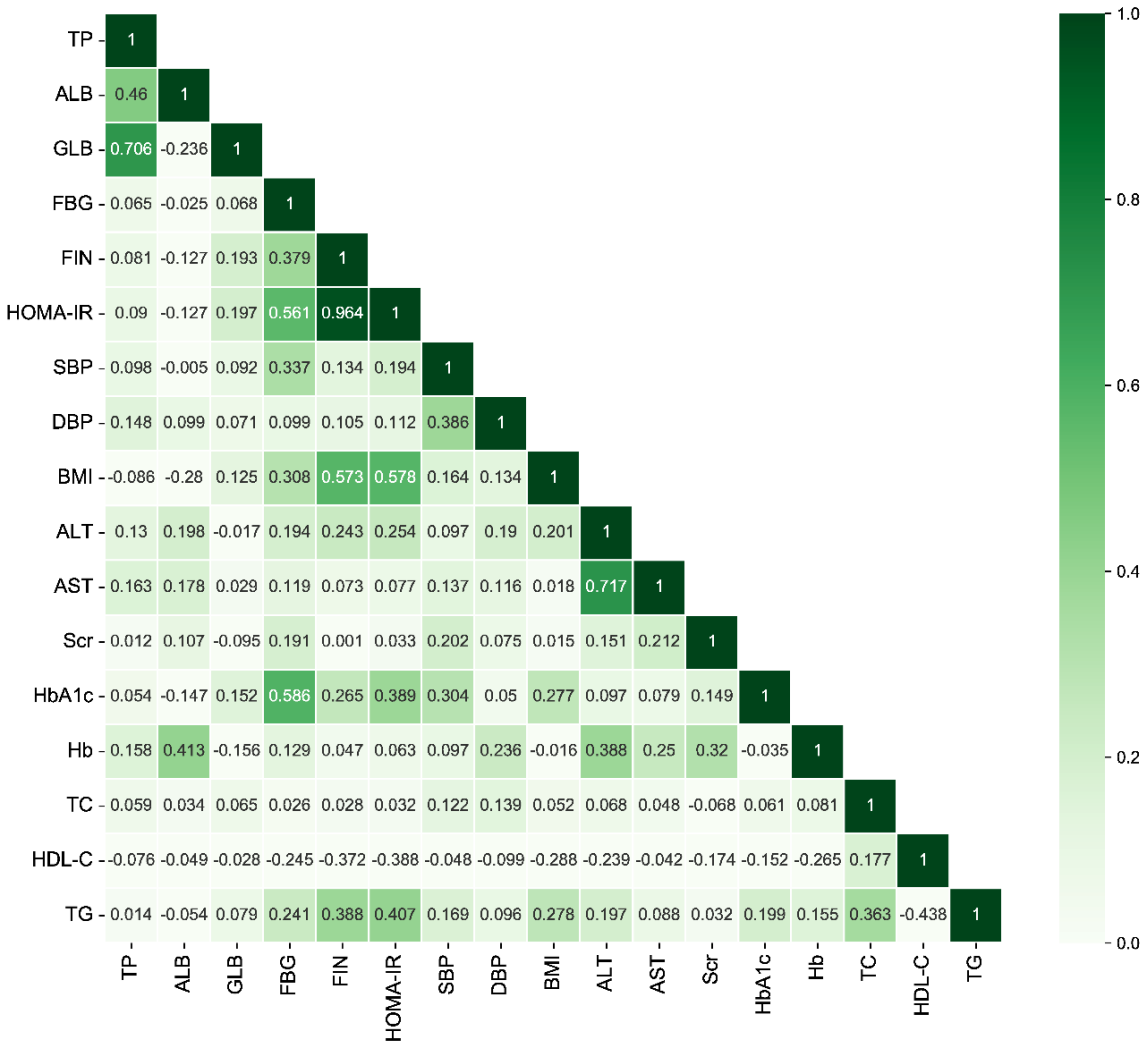
The heatmap of the correlation between covariates using Spearman’s correlation analysis. TP, total protein; ALB, albumin; GLB, globulin; FBG, fasting blood-glucose; FIN, fasting insulin; HOMA-IR, homeostasis model assessment of insulin resistance; SBP, systolic blood pressure; DBP, diastolic blood pressure; BMI, body mass index; ALT, alanine aminotransferase; AST, aspartate aminotransferase; Scr, serum creatinine; HbA1c, glycated hemoglobin A1c; Hb, hemoglobin; TC, total cholesterol; HDL-C, high-density lipoprotein cholesterol; TG, triglycerides.

The relations between baseline variables and HOMA-IR were assessed using Spearman’s correlation analysis (**Figure 3**, **Supplementary Tables S1**). HOMA-IR was mostly positively associated with TP, GLB, FBG, FIN, SBP, DBP, BMI, ALT, AST, Scr, HbA1c, Hb, TC, and TG. However, an inverse correlation was found between ALB and HDL-C.

**Figure 4:**
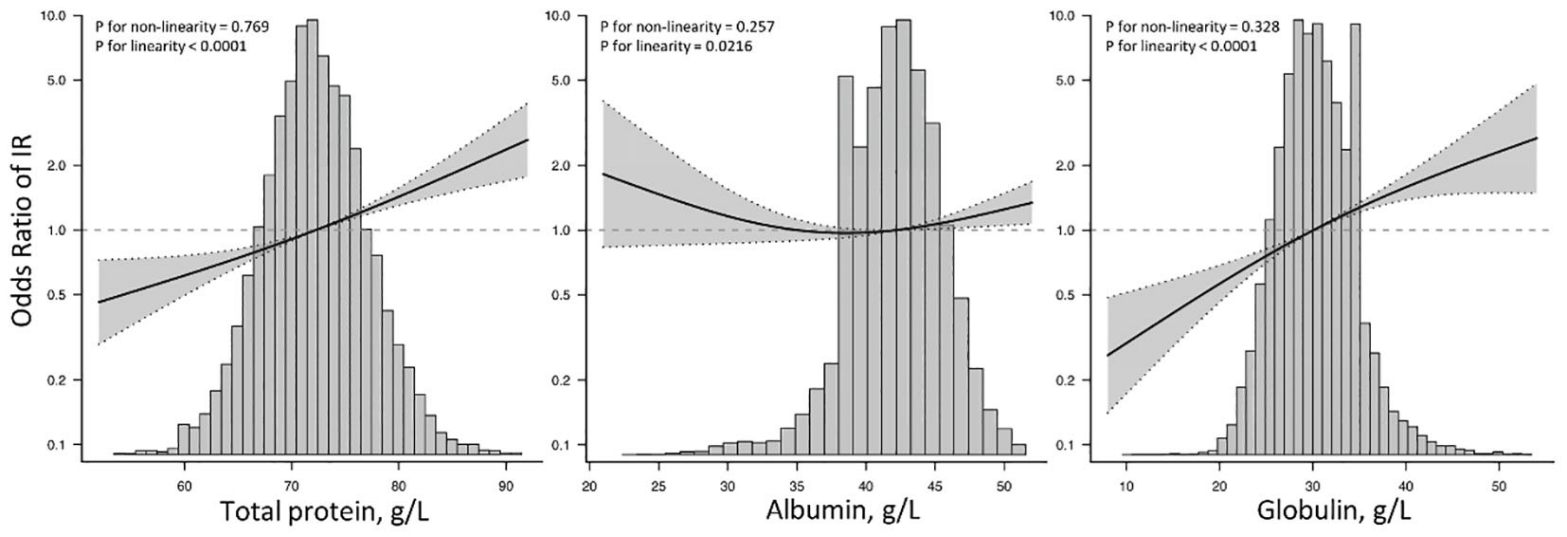
Restricted cubic spine models of TP, ALB, and GLB with insulin resistance. TP, total protein; ALB, albumin; GLB, globulin; IR, insulin resistance. Restricted cubic spine models were adjusted for age, gender, race, education, systolic blood pressure, diastolic blood pressure, body mass index, smoking, diabetes, hypertension, coronary heart disease, acute myocardial infarction, chronic heart failure, stroke, cancer, alanine aminotransferase, aspartate aminotransferase, serum creatinine, glycated hemoglobin A1c, hemoglobin, total cholesterol, high-density lipoprotein cholesterol, and triglycerides.

Furthermore, as demonstrated in **Figure 4**, the multivariate-adjusted restrictive cubic curves further confirmed that the relationships of TP and GLB with IR prevalence were linear.

### Subgroup analysis of the risk of incident IR

**Figure 5:**
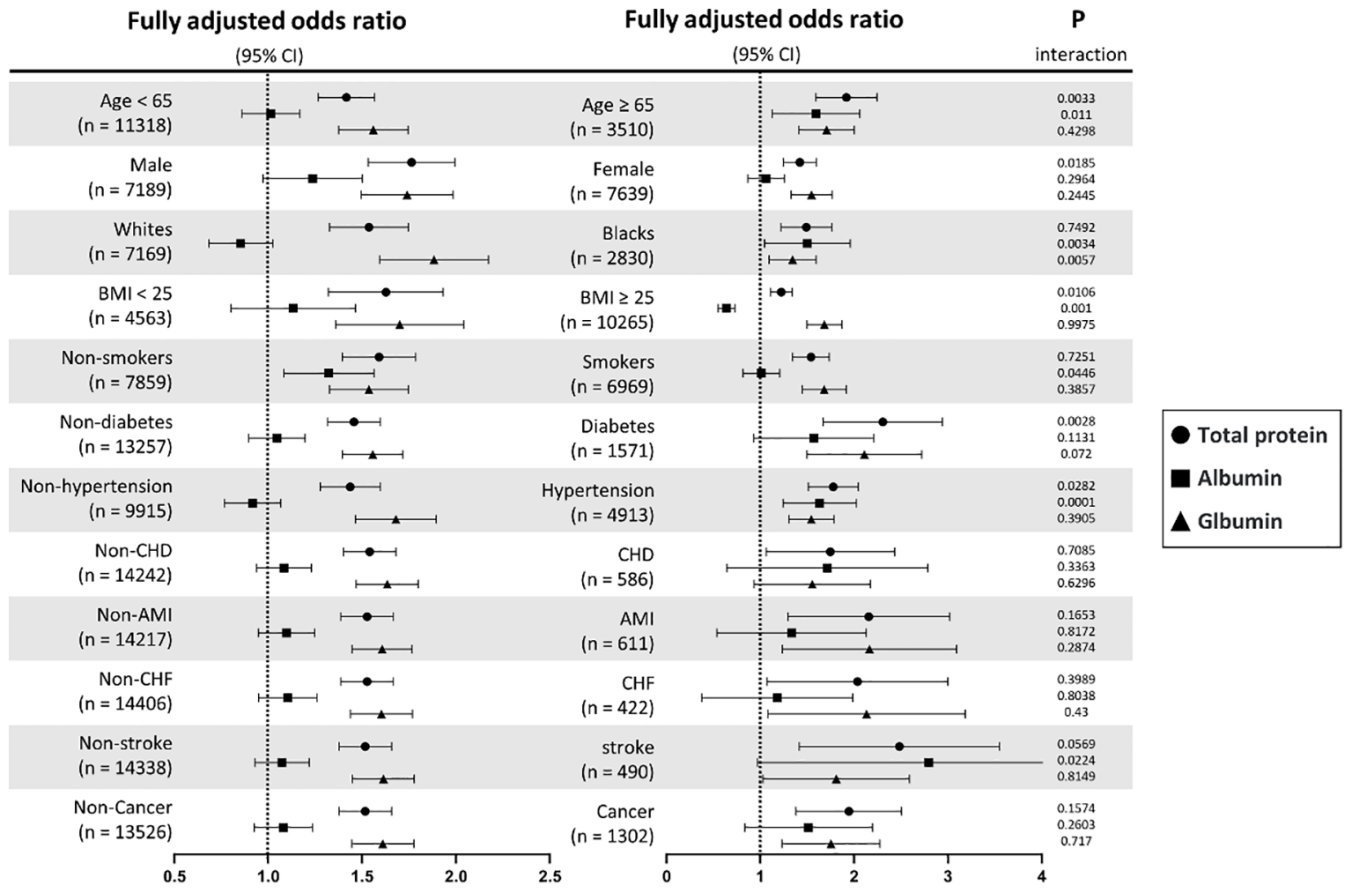
Subgroup analyses of TP, ALB, and GLB with insulin resistance stratified by participant characteristics. TP, total protein; ALB, albumin; GLB, globulin; BMI, body mass index; CHD, coronary heart disease; AMI, acute myocardial infarction; CHF, chronic heart failure. Results are expressed as multivariable-adjusted odds ratio in continuous analyses (per 10g/L increment) after controlling covariates including age, gender, race, education, systolic blood pressure, diastolic blood pressure, body mass index, smoking, diabetes, hypertension, coronary heart disease, acute myocardial infarction, chronic heart failure, stroke, cancer, alanine aminotransferase, aspartate aminotransferase, serum creatinine, glycated hemoglobin A1c, hemoglobin, total cholesterol, high-density lipoprotein cholesterol, and triglycerides, where possible interactions between above factors are also adjusted for if necessary.

The stratified analyses performed using multivariate logistic regression analysis and interactions analysis are shown in **Figure 5** and **Supplementary Tables S2**. The association between TP and IR risks was generally significant across all subgroups. For ALB, the significant associations were only found among participants who were aged ≥ 65 years, Black persons, those with a BMI ≥ 25 kg/m^2^, non-smokers, and those with hypertension. Furthermore, the positive association between GLB and IR risks was found in general subgroups, but not in patients with CHD.

## Discussion

This study contributes novel evidence indicating that both TP and GLB exhibit strong linear relationships with increased incident risk of IR, whereas no significant association was observed with ALB. The positive correlation between ALB and IR prevalence could be observed in older persons, Black persons, and those with hypertension, and a negative correlation was only found in those with a BMI ≥ 25 kg/m^2^. These findings suggest potential clinical implications for a deeper understanding of the impact of TP, ALB, and GLB on IR.

Insulin resistance, a key driver of various metabolic-related diseases, describes the reduced responsiveness of insulin-targeted tissues to elevated insulin levels (16). Prior research has indicated an association between serum ALB and IR, possibly attributable to the theory that compensatory hyperinsulinemia in cases of IR promotes ALB synthesis. Experts have demonstrated that serum insulin positively regulates ALB gene transcription and mRNA synthesis in rats *in vivo* and *in vitro* (17–19). A similar trend was also observed in patients with type 1 diabetes (20). However, in the atherogenic process, serum ALB is negatively correlated with inflammation and oxidative stress, which is considered to play crucial roles in the prevention and treatment of IR (6, 21). However, in our study, this protective effect was not observed. Furthermore, as is well known, TP and GLB levels in patients provide valuable insights into their overall health status. TP reflects the body’s nutritional status, liver function, kidney function, and the presence of chronic inflammatory or infectious diseases (22, 23). GLB, a component of TP, plays a critical role in immune response and protein transport. Elevated GLB levels may indicate chronic inflammatory conditions, liver disease, or immune disorders, while low levels can suggest malnutrition or protein synthesis issues (24–26). However, there is currently no existing evidence supporting an association of TP or GLB with IR. Thus, further basic and clinical research is necessary to clarify the underlying mechanism and validate these findings.

To our knowledge, this is the first report with a large sample size aimed at comprehensively understanding the associations between serum proteins, including TP, ALB, and GLB, and IR within the same population. The conclusion is supported by available robust clinical data from NHANES, including FGB, FIN, and multiple traditional IR risks. Previously, Ji et al. revealed a positive association between ALB and IR among 9,029 subjects without diabetes in Korea. However, they did not report a correlation between the other two serum proteins and IR (7). Compared to the study by Ji et al., we further determined a detailed relationship of TP, ALB, and GLB with Incident IR with a larger sample, broader population, and more comprehensive adjustments. It was confirmed that the values of TP and GLB were positively correlated with IR. However, in our study, the significant associations between ALB and IR were only found among participants who were aged ≥ 65 years, Black persons, those with a BMI ≥ 25 kg/m^2^, non-smokers, and those with hypertension. The discrepancy between the results of Ji et al. and ours is probably attributable to differences in the study population, age distribution, and dietary patterns. Regarding clinical importance, our novel findings are conducive to understanding the relationship between serum proteins and IR and remind us that when evaluating the risk of patients with possible IR, attention should also be paid to the serum protein concentrations, rather than solely on changes in blood-glucose indexes.

However, the present study still has several limitations. First, given the nature of observational cross-sectional studies, each serum protein concentration can only be measured once, leading to potential bias, and no causal relationship between any serum protein concentration and IR prevalence can be drawn. Second, the present study is based on data from the NHANES study, conducted by the United States National Center, making the conclusion difficult to extrapolate to other regions of the world. Therefore, this conclusion still needs to be interpreted with caution.

## Conclusions

The present cross-sectional study with a large sample size provided supportive evidence of positive associations of TP and GLB with IR. Additionally, a linear association between ALB and the prevalence of IR could be observed in older persons, Black persons, and participants with hypertension, and a negative correlation was only found in those with a BMI ≥ 25 kg/m^2^. The association between serum proteins and IR suggests the necessity of introducing serum protein monitoring into the management of patients with possible IR and further well-designed multicenter prospective studies are necessary to determine the specific effects of these serum proteins on IR in humans.

## Data Availability

The datasets presented in this study can be found in online repositories. The names of the repository/repositories and accession number(s) can be found in the article/**Supplementary Material**.

## References

[1] Lebovitz HE. Insulin resistance: definition and consequences. Exp Clin Endocrinol Diabetes. (2001) 109 Suppl 2:S135–48. doi: 10.1055/s-2001-18576 11460565

[2] Lee SH Park SY Choi CS. Insulin resistance: from mechanisms to therapeutic strategies. Diabetes Metab J. (2022) 46:15–37. doi: 10.4093/dmj.2021.0280 34965646 PMC8831809

[3] Adnan E Rahman IA Faridin HP. Relationship between insulin resistance, metabolic syndrome components and serum uric acid. Diabetes Metab Syndr. (2019) 13:2158–62. doi: 10.1016/j.dsx.2019.04.001 31235151

[4] Malik SUF Mahmud Z Alam J Islam MS Azad AK. Relationship among obesity, blood lipids and insulin resistance in Bangladeshi adults. Diabetes Metab Syndr. (2019) 13:444–9. doi: 10.1016/j.dsx.2018.10.015 30641741

[5] Adeva-Andany MM Martinez-Rodriguez J Gonzalez-Lucan M Fernandez-Fernandez C Castro-Quintela E. Insulin resistance is a cardiovascular risk factor in humans. Diabetes Metab Syndr. (2019) 13:1449–55. doi: 10.1016/j.dsx.2019.02.023 31336505

[6] Ishizaka N Ishizaka Y Nagai R Toda E Hashimoto H Yamakado M. Association between serum albumin, carotid atherosclerosis, and metabolic syndrome in Japanese individuals. Atherosclerosis. (2007) 193:373–9. doi: 10.1016/j.atherosclerosis.2006.06.031 16904116

[7] Bae JC Seo SH Hur KY Kim JH Lee MS Lee MK. Association between serum albumin, insulin resistance, and incident diabetes in nondiabetic subjects. Endocrinol Metab (Seoul). (2013) 28:26–32. doi: 10.3803/EnM.2013.28.1.26 24396647 PMC3811792

[8] Danesh J Collins R Appleby P Peto R. Association of fibrinogen, C-reactive protein, albumin, or leukocyte count with coronary heart disease: meta-analyses of prospective studies. JAMA. (1998) 279:1477–82. doi: 10.1001/jama.279.18.1477 9600484

[9] Djousse L Rothman KJ Cupples LA Levy D Ellison RC. Serum albumin and risk of myocardial infarction and all-cause mortality in the Framingham Offspring Study. Circulation. (2002) 106:2919–24. doi: 10.1161/01.cir.0000042673.07632.76 12460872

[10] Ford ES Giles WH Dietz WH. Prevalence of the metabolic syndrome among US adults: findings from the third National Health and Nutrition Examination Survey. JAMA. (2002) 287:356–9. doi: 10.1001/jama.287.3.356 11790215

[11] Palmer MK Toth PP. Trends in lipids, obesity, metabolic syndrome, and diabetes mellitus in the United States: an NHANES analysis (2003-2004 to 2013-2014). Obes (Silver Spring). (2019) 27:309–14. doi: 10.1002/oby.22370 30677260

[12] Qu HQ Li Q Lu Y Hanis CL Fisher-Hoch SP McCormick JB. Ancestral effect on HOMA-IR levels quantitated in an American population of Mexican origin. Diabetes Care. (2012) 35:2591–3. doi: 10.2337/dc12-0636 PMC3507582 22891255

[13] Sumner AE Cowie CC. Ethnic differences in the ability of triglyceride levels to identify insulin resistance. Atherosclerosis. (2008) 196:696–703. doi: 10.1016/j.atherosclerosis.2006.12.018 17254586

[14] Voruganti VS Lopez-Alvarenga JC Nath SD Rainwater DL Bauer R Cole SA. Genetics of variation in HOMA-IR and cardiovascular risk factors in Mexican-Americans. J Mol Med (Berl). (2008) 86:303–11. doi: 10.1007/s00109-007-0273-3 18204828

[15] Matthews DR Hosker JP Rudenski AS Naylor BA Treacher DF Turner RC. Homeostasis model assessment: insulin resistance and beta-cell function from fasting plasma glucose and insulin concentrations in man. Diabetologia. (1985) 28:412–9. doi: 10.1007/BF00280883 3899825

[16] Petersen MC Shulman GI. Mechanisms of insulin action and insulin resistance. Physiol Rev. (2018) 98:2133–223. doi: 10.1152/physrev.00063.2017 PMC6170977 30067154

[17] Kimball SR Horetsky RL Jefferson LS. Hormonal regulation of albumin gene expression in primary cultures of rat hepatocytes. Am J Physiol. (1995) 268:E6–14. doi: 10.1152/ajpendo.1995.268.1.E6 7840183

[18] Lloyd CE Kalinyak JE Hutson SM Jefferson LS. Stimulation of albumin gene transcription by insulin in primary cultures of rat hepatocytes. Am J Physiol. (1987) 252:C205–14. doi: 10.1152/ajpcell.1987.252.2.C205 3548414

[19] Peavy DE Taylor JM Jefferson LS. Time course of changes in albumin synthesis and mRNA in diabetic and insulin-treated diabetic rats. Am J Physiol. (1985) 248:E656–63. doi: 10.1152/ajpendo.1985.248.6.E656 3890555

[20] De Feo P Gaisano MG Haymond MW. Differential effects of insulin deficiency on albumin and fibrinogen synthesis in humans. J Clin Invest. (1991) 88:833–40. doi: 10.1172/JCI115384 PMC295469 1909352

[21] Andreadi A Bellia A Di Daniele N Meloni M Lauro R Della-Morte D. The molecular link between oxidative stress, insulin resistance, and type 2 diabetes: A target for new therapies against cardiovascular diseases. Curr Opin Pharmacol. (2022) 62:85–96. doi: 10.1016/j.coph.2021.11.010 34959126

[22] Patton HM. Nutritional assessment of patients with chronic liver disease. Gastroenterol Hepatol (N Y). (2012) 8:687–90. PMC3969013 24683378

[23] Zha Y Qian Q. Protein nutrition and malnutrition in CKD and ESRD. Nutrients. (2017) 9. doi: 10.3390/nu9030208 PMC5372871 28264439

[24] Hashash JG Koutroumpakis F Anderson AM Rivers CR Hosni M Koutroubakis IE. Elevated serum globulin fraction as a biomarker of multiyear disease severity in inflammatory bowel disease. Ann Gastroenterol. (2022) 35:609–17. doi: 10.20524/aog.2022.0748 PMC9648529 36406970

[25] Liu C Wang W Meng X Sun B Cong Y Liu J. Albumin/globulin ratio is negatively correlated with PD-1 and CD25 mRNA levels in breast cancer patients. Onco Targets Ther. (2018) 11:2131–9. doi: 10.2147/OTT.S159481 PMC5905531 29899663

[26] Wang J Liu F Kong R Han X. Association between globulin and diabetic nephropathy in type2 diabetes mellitus patients: A cross-sectional study. Front Endocrinol (Lausanne). (2022) 13:890273. doi: 10.3389/fendo.2022.890273 35898464 PMC9311329

