# Supplementary material for: Associations of total protein, albumin, and globulin with insulin resistance: an NHANES study
Source: Front Endocrinol (Lausanne). 2024 Sep 13;15:1393137. doi: 10.3389/fendo.2024.1393137 (PMC11427264; doi:10.3389/fendo.2024.1393137)
Supplement: Supplementary file 1 [file Table1.docx]

**TABEL S1** Bivariate associations between baseline variables.

|  | **TP** | **ALB** | **GLB** | **FBG** | **FIN** | **HOMA-IR** | **SBP** | **DBP** | **BMI** | **ALT** | **AST** | **Scr** | **HbA1c** | **Hb** | **TC** | **HDL-C** | **TG** |
| --- | --- | --- | --- | --- | --- | --- | --- | --- | --- | --- | --- | --- | --- | --- | --- | --- | --- |
| **TP** | 1 | 0.4604 | 0.7055 | 0.0645 | 0.0814 | 0.09 | 0.0979 | 0.1482 | -0.0862 | 0.1298 | 0.1632 | 0.0124 | 0.0538 | 0.1578 | 0.0585 | -0.0763 | 0.014 |
| **ALB** | 0.4604 | 1 | -0.2358 | -0.025 | -0.127 | -0.1265 | -0.0053 | 0.0988 | -0.2798 | 0.1983 | 0.178 | 0.1071 | -0.1472 | 0.4132 | 0.0342 | -0.0485 | -0.0536 |
| **GLB** | 0.7055 | -0.2358 | 1 | 0.0683 | 0.1931 | 0.1967 | 0.092 | 0.0711 | 0.1247 | -0.017 | 0.0288 | -0.0947 | 0.1519 | -0.1555 | 0.0653 | -0.0278 | 0.0793 |
| **FBG** | 0.0645 | -0.025 | 0.0683 | 1 | 0.3788 | 0.561 | 0.3365 | 0.0988 | 0.3081 | 0.1941 | 0.1186 | 0.1912 | 0.5859 | 0.1291 | 0.0262 | -0.2453 | 0.2406 |
| **FIN** | 0.0814 | -0.127 | 0.1931 | 0.3788 | 1 | 0.9643 | 0.1342 | 0.105 | 0.5726 | 0.2426 | 0.0729 | 0.0006 | 0.2646 | 0.047 | 0.0282 | -0.3715 | 0.3876 |
| **HOMA-IR** | 0.09 | -0.1265 | 0.1967 | 0.561 | 0.9643 | 1 | 0.194 | 0.1116 | 0.5782 | 0.2536 | 0.0771 | 0.0331 | 0.3885 | 0.0627 | 0.0324 | -0.3875 | 0.4068 |
| **SBP** | 0.0979 | -0.0053 | 0.092 | 0.3365 | 0.1342 | 0.194 | 1 | 0.3857 | 0.1636 | 0.0965 | 0.1373 | 0.202 | 0.3037 | 0.0965 | 0.1218 | -0.0484 | 0.1693 |
| **DBP** | 0.1482 | 0.0988 | 0.0711 | 0.0988 | 0.105 | 0.1116 | 0.3857 | 1 | 0.1337 | 0.1902 | 0.1163 | 0.0749 | 0.0501 | 0.2357 | 0.1391 | -0.0991 | 0.0963 |
| **BMI** | -0.0862 | -0.2798 | 0.1247 | 0.3081 | 0.5726 | 0.5782 | 0.1636 | 0.1337 | 1 | 0.2007 | 0.0175 | 0.0151 | 0.2773 | -0.016 | 0.0523 | -0.2875 | 0.2779 |
| **ALT** | 0.1298 | 0.1983 | -0.017 | 0.1941 | 0.2426 | 0.2536 | 0.0965 | 0.1902 | 0.2007 | 1 | 0.7171 | 0.1513 | 0.0972 | 0.3881 | 0.0684 | -0.2389 | 0.1965 |
| **AST** | 0.1632 | 0.178 | 0.0288 | 0.1186 | 0.0729 | 0.0771 | 0.1373 | 0.1163 | 0.0175 | 0.7171 | 1 | 0.2116 | 0.079 | 0.2496 | 0.0482 | -0.0422 | 0.0876 |
| **Scr** | 0.0124 | 0.1071 | -0.0947 | 0.1912 | 0.0006 | 0.0331 | 0.202 | 0.0749 | 0.0151 | 0.1513 | 0.2116 | 1 | 0.1493 | 0.3199 | -0.068 | -0.1737 | 0.0318 |
| **HbA1c** | 0.0538 | -0.1472 | 0.1519 | 0.5859 | 0.2646 | 0.3885 | 0.3037 | 0.0501 | 0.2773 | 0.0972 | 0.079 | 0.1493 | 1 | -0.0348 | 0.0606 | -0.1519 | 0.1993 |
| **Hb** | 0.1578 | 0.4132 | -0.1555 | 0.1291 | 0.047 | 0.0627 | 0.0965 | 0.2357 | -0.016 | 0.3881 | 0.2496 | 0.3199 | -0.0348 | 1 | 0.0812 | -0.265 | 0.1551 |
| **TC** | 0.0585 | 0.0342 | 0.0653 | 0.0262 | 0.0282 | 0.0324 | 0.1218 | 0.1391 | 0.0523 | 0.0684 | 0.0482 | -0.068 | 0.0606 | 0.0812 | 1 | 0.1769 | 0.3627 |
| **HDL-C** | -0.0763 | -0.0485 | -0.0278 | -0.2453 | -0.3715 | -0.3875 | -0.0484 | -0.0991 | -0.2875 | -0.2389 | -0.0422 | -0.1737 | -0.1519 | -0.265 | 0.1769 | 1 | -0.4383 |
| **TG** | 0.014 | -0.0536 | 0.0793 | 0.2406 | 0.3876 | 0.4068 | 0.1693 | 0.0963 | 0.2779 | 0.1965 | 0.0876 | 0.0318 | 0.1993 | 0.1551 | 0.3627 | -0.4383 | 1 |

TP, total protein; ALB, albumin; GLB, Globulin; FBG, fasting blood-glucose; FIN, fasting insulin; HOMA-IR, homeostasis model assessment of insulin resistance; SBP, systolic blood pressure; DBP, diastolic blood pressure; BMI, body mass index; ALT, alanine aminotransferase; AST, aspartate aminotransferase; Scr, serum creatinine; HbA1c, glycated hemoglobin A1c; Hb, hemoglobin; TC, total cholesterol; HDL-C, high-density lipoprotein cholesterol; TG, triglycerides.

**TABEL S2** Subgroup analyses of TP, ALB, and GLB with insulin resistance stratified by participant characteristics.

|  | **Fully adjusted odds ratio (95% CI), P-value** | | P interaction |
| --- | --- | --- | --- |
|  | **Age < 65 (n = 11318)** | **Age ≥ 65 (n = 3510)** |  |
| TP | 1.41 (1.27, 1.57) <0.0001 | 1.90 (1.60, 2.25) <0.0001 | 0.0033 |
| ALB | 1.01 (0.86, 1.17) 0.9377 | 1.55 (1.15, 2.08) 0.0037 | 0.011 |
| GLB | 1.55 (1.38, 1.75) <0.0001 | 1.69 (1.42, 2.01) <0.0001 | 0.4298 |
|  | **Male (n = 7189)** | **Female (n = 7639)** |  |
| TP | 1.75 (1.54, 2.00) <0.0001 | 1.41 (1.25, 1.60) <0.0001 | 0.0185 |
| ALB | 1.22 (0.98, 1.51) 0.0738 | 1.05 (0.87, 1.26) 0.6141 | 0.2964 |
| GLB | 1.73 (1.50, 1.99) <0.0001 | 1.54 (1.33, 1.77) <0.0001 | 0.2445 |
|  | **Whites (n = 7169)** | **Blacks (n = 2830)** |  |
| TP | 1.53 (1.33, 1.75) <0.0001 | 1.47 (1.23, 1.77) <0.0001 | 0.7492 |
| ALB | 0.84 (0.69, 1.03) 0.0962 | 1.45 (1.07, 1.98) 0.0171 | 0.0034 |
| GLB | 1.87 (1.60, 2.18) <0.0001 | 1.33 (1.10, 1.60) 0.0036 | 0.0057 |
|  | **BMI < 25 (n = 4563)** | **BMI ≥ 25 (n = 10265)** |  |
| TP | 1.61 (1.33, 1.94) <0.0001 | 1.22 (1.11, 1.34) <0.0001 | 0.0106 |
| ALB | 1.10 (0.82, 1.48) 0.5287 | 0.63 (0.55, 0.73) <0.0001 | 0.001 |
| GLB | 1.68 (1.37, 2.05) <0.0001 | 1.68 (1.50, 1.87) <0.0001 | 0.9975 |
|  | **Non-smokers (n = 7859)** | **Smokers (n = 6969)** |  |
| TP | 1.58 (1.40, 1.79) <0.0001 | 1.53 (1.35, 1.74) <0.0001 | 0.7251 |
| ALB | 1.31 (1.09, 1.57) 0.0044 | 1.00 (0.82, 1.21) 0.9770 | 0.0446 |
| GLB | 1.53 (1.33, 1.75) <0.0001 | 1.67 (1.45, 1.92) <0.0001 | 0.3857 |
|  | **Non-diabetes (n = 13257)** | **Diabetes (n = 1571)** |  |
| TP | 1.45 (1.32, 1.60) <0.0001 | 2.25 (1.70, 2.97) <0.0001 | 0.0028 |
| ALB | 1.04 (0.90, 1.20) 0.6019 | 1.48 (0.98, 2.25) 0.0634 | 0.1131 |
| GLB | 1.55 (1.40, 1.72) <0.0001 | 2.05 (1.53, 2.75) <0.0001 | 0.072 |
|  | **Non-hypertension (n = 9915)** | **Hypertension (n = 4913)** |  |
| TP | 1.43 (1.28, 1.60) <0.0001 | 1.76 (1.52, 2.05) <0.0001 | 0.0282 |
| ALB | 0.91 (0.77, 1.07) 0.2603 | 1.60 (1.26, 2.04) 0.0001 | 0.0001 |
| GLB | 1.67 (1.47, 1.90) <0.0001 | 1.53 (1.31, 1.79) <0.0001 | 0.3905 |
|  | **Non-CHD (n = 14242)** | **CHD (n = 586)** |  |
| TP | 1.54 (1.40, 1.68) <0.0001 | 1.66 (1.11, 2.47) 0.0128 | 0.7085 |
| ALB | 1.08 (0.94, 1.23) 0.3046 | 1.49 (0.77, 2.88) 0.2310 | 0.3363 |
| GLB | 1.63 (1.47, 1.80) <0.0001 | 1.47 (0.98, 2.21) 0.0647 | 0.6296 |
|  | **Non-AMI (n = 14217)** | **AMI (n = 611)** |  |
| TP | 1.52 (1.39, 1.67) <0.0001 | 2.04 (1.36, 3.07) 0.0006 | 0.1653 |
| ALB | 1.09 (0.95, 1.25) 0.2335 | 1.17 (0.63, 2.20) 0.6181 | 0.8172 |
| GLB | 1.60 (1.45, 1.77) <0.0001 | 2.03 (1.31, 3.15) 0.0014 | 0.2874 |
|  | **Non-CHF (n = 14406)** | **CHF (n = 422)** |  |
| TP | 1.52 (1.39, 1.67) <0.0001 | 1.88 (1.16, 3.07) 0.0108 | 0.3989 |
| ALB | 1.10 (0.95, 1.26) 0.1919 | 1.00 (0.48, 2.06) 0.9972 | 0.8038 |
| GLB | 1.60 (1.44, 1.77) <0.0001 | 1.96 (1.18, 3.26) 0.0089 | 0.43 |
|  | **Non-stroke (n = 14338)** | **Stroke (n = 490)** |  |
| TP | 1.51 (1.38, 1.66) <0.0001 | 2.33 (1.50, 3.62) 0.0002 | 0.0569 |
| ALB | 1.07 (0.93, 1.22) 0.3543 | 2.40 (1.20, 4.79) 0.0132 | 0.0224 |
| GLB | 1.61 (1.45, 1.78) <0.0001 | 1.70 (1.09, 2.64) 0.0196 | 0.8149 |
|  | **Non-Cancer (n = 13526)** | **Cancer (n = 1302)** |  |
| TP | 1.51 (1.38, 1.66) <0.0001 | 1.89 (1.41, 2.53) <0.0001 | 0.1574 |
| ALB | 1.07 (0.93, 1.24) 0.3271 | 1.41 (0.89, 2.24) 0.1411 | 0.2603 |
| GLB | 1.60 (1.45, 1.78) <0.0001 | 1.70 (1.26, 2.30) 0.0006 | 0.717 |

TP, total protein; ALB, albumin; GLB, Globulin; BMI, body mass index; CHD, coronary heart disease; AMI, acute myocardial infarction; CHF, chronic heart failure.

Results are expressed as multivariable-adjusted odds ratio in continuous analyses (per 10g/L increment) after controlling covariates including age, gender, race, education, systolic blood pressure, diastolic blood pressure, body mass index, smoking, diabetes, hypertension, CHD, AMI, CHF, stroke, cancer, alanine aminotransferase, aspartate aminotransferase, serum creatinine, glycated hemoglobin A1c, hemoglobin, total cholesterol, high-density lipoprotein cholesterol and triglycerides, where possible interactions between above factors are also adjusted if necessary.
